# Distrust and reflexive impotence in the net zero transition: findings from a longitudinal deliberative mini-public

**DOI:** 10.1007/s10584-024-03806-2

**Published:** 2024-10-28

**Authors:** Jacob Ainscough, Pancho Lewis, Lucy Farrow

**Affiliations:** 1 Lancaster Environment Centre, Lancaster University, Lancaster, UK; 2 Thinks Insights, London, UK

**Keywords:** Public engagement, Public attitudes, Climate citizenship, Deliberative mini-publics

## Abstract

Responding to climate change requires that people engage in different forms of climate citizenship. These span from individual consumption choices, to taking part in forms of collective action to steer the behaviour of governments and in the private sector. Here we analyse data from the Net Zero Diaries project to explore how attitudes to different forms of climate citizenship develop as people become more aware about the scale of societal change required to reach net zero carbon emissions by 2050. The Net Zero Diaries project was a deliberative mini-public, composed of 41 citizens broadly representative of the UK adult population, which convened over five months between 2021 and 2022 to debate the UK policies for reaching net zero emissions by 2050. We show that people identify government as the prime actor needed to drive the transition, but doubt that they will act due to a range of constraints vis-à-vis the public and private sector. This tension provides a novel explanation for why some people prioritise forms of individual and consumption focused climate citizenship, whilst also doubting the efficacy of such actions. We conclude by suggesting that efforts to drive more engaged forms of climate citizenship need to attend to underlying feelings about state efficacy, rather than focus on just how the issue of climate change is framed.

**Supplementary Information:**

The online version contains supplementary material available at 10.1007/s10584-024-03806-2.

## Introduction

In order to transition to a net zero society, people will need to make significant changes to how they live their lives. This includes changes to diets, travel, how homes are heated, and in some cases to the jobs that people do (Climate Change Committee 2020). But ensuring such changes happen in a democratic fashion and without societal kickback requires forms of ‘climate citizenship’ beyond adjustments in individual consumption (Willis 2020) – from small-scale, community-focused activities through to action directed at large-scale, political reform. With respect to localised action, engaging in conversations about climate change with friends, neighbours and colleagues can help to spread climate change awareness and shift societal norms (Sparkman et al. 2020). Similarly, getting involved in community initiatives – such as alternative food networks and community energy groups – can contribute to decarbonising certain sectors (Warren and McFadyen 2010). With regards to large-scale transformations, citizen lobbying of politicians is necessary to drive the transition. In addition, there needs to be consensus building among publics so that pressure on policy decision-makers pursuing rapid decarbonisation is maintained (Willis 2020).

The degree to which people engage in different forms of climate citizenship is in part shaped by their cognitive and affective responses to the threat of climate change (Marlon et al. 2019; Ojala 2023). Ample research has shown that awareness of climate change generates different emotional responses, including feelings of hope (Ojala 2012, 2023), worry (Smith and Leiserowitz 2014; Stevenson et al. 2017) and boredom (Anderson 2021), all of which may encourage or stymie engagement and action on climate.

As of spring 2023, 82% of UK adults reported being fairly or very concerned about climate change (UK Government 2023). Such high levels of concern are now the norm across much of the world (Flynn and Yamasumi 2021). Yet a much smaller proportion of the UK population, approximately 13%, are politically active in advancing the cause of carbon mitigation (Britain Talks Climate 2020). Thus, there is a gap between levels of climate concern, and people urging political actors to take more concerted action. Most people tend to limit their support to climate policies that do not require them to make significant lifestyle changes and will not lead to additional costs in their lives (Britain Talks Climate 2020; Ipsos and CAST 2022).

There is an additional dimension to add to this picture. Though there is growing concern about climate change, many people do not grasp the scale of societal transformation required to achieve net zero emissions. In the UK, as in other countries, carbon mitigation to date has resulted from changes that have rarely affected people’s lives. As governments start to address emissions from sectors such as housing, food and agriculture, and transport, people will necessarily become more aware of the scale of transformation required to reach net zero. This raises an important set of questions. How will publics respond to these changes? Will people come to practise more active forms of citizenship, where they engage in dialogue with government to make climate policy ‘work’ for their lives? Or will adopt a more passive role, consenting to policies without actively responding to them? Conversely, will they become increasingly resistant to the types of changes being demanded of them and push back?

### Aims of the research

This research explores how learning about the policy changes needed to reach climate goals affects people’s disposition towards different forms of climate citizenship. In particular, the paper examines the arguments and lines of reasoning used by citizens in developing their views about the value of different forms of climate citizenship. By studying discussions among a small group of people, specifically a ‘deliberative-mini public’ (see below), we aim to identify potential issues that may arise as the wider public becomes more aware of the policy challenges of climate change. In addition, we identify strategies for supporting forms of climate citizenship more conducive to achieving a democratic transition to a low carbon economy.

### Deliberative mini-publics as laboratories of ‘becoming aware’

One way to explore what learning about the challenges of achieving net zero emissions does to attitudes towards climate citizenship is within deliberative mini-public (from here-on referred to as ‘DMP’) (Willis et al. 2022). DMPs facilitate learning and dialogue between a group of demographically representative citizens, allowing them to debate an issue of mutual concern. People typically leave DMPs feeling much more informed about the issue at hand (Elstub et al. 2021, Andrews et al. 2022). Participation in a DMP also impacts on people’s attitudes and behaviours beyond the lifespan of the process itself (van der Does and Jacquet 2021). Thus, learning that occurs within DMPs meaningfully impacts how people choose to live in the world. Though not a perfect analogue to real world learning, climate focused DMPs thus provide a valuable setting within which to explore the coevolution of understanding, affective responses, and attitudes towards climate citizenship.

##  Method

### Net zero diaries

The data for this project were collected through a deliberative project called ‘Net Zero Diaries’ (NZD). The authors of this paper ran the project with BritainThinks (now renamed as ‘Thinks Insights’) a public engagement consultancy who co-led the design and management of the process. The aim of NZD was to study the evolution of public attitudes to climate policy in ‘real time’ as political events unfolded during the UNFCC’s 26th Conference of the Parties (COP) in Glasgow.^1^ We tracked how attitudes changed in three stages – in the run up to, during, and after the event.

Participants were recruited through a purposive sampling approach to be broadly demographically representative of the UK adult population in terms of gender, age, housing tenure, ethnicity, and health status. In addition, we recruited people according to one of three categories: people who had previously taken part in a DMP on climate policy (labelled ‘engaged citizens’ for the sake of the study^2^); people who had carried out significant changes in their lives to reduce their carbon footprint, such as buying an electric car or installing an electric heat pump (labelled ‘engaged consumers’); and the general public (i.e., people who had neither taken part in a climate DMP or made changes to reduce their carbon footprint). The total sample consisted of seven engaged citizens, 19 engaged consumers, and 15 members of the general public. Differences between these sub-groups were tracked through the process and are reported here when relevant. All participants were given a financial incentive to take part in the research (see the Supplementary Material for a summary of the demographics of the participants).

The research took place in four waves between September 2021 and January 2022. Each wave consisted of five days of online activities. These included answering polls and open text questions about attitudes and behaviours, making use of prompt material from recent climate news and current affairs. At the end of each five-day period, the participants met for a full-day online deliberative workshop (see the Supplementary Material for details of the themes and core activities of each workshop. A code is assigned to each workshop session for the purpose of referencing them in the results section).

The workshops at the end of each wave of research were structured in a similar way to standard DMPs, with participants receiving expert presentations, being given the chance to ask questions of experts, and having time to deliberate amongst themselves over different elements of the net zero transition. The workshops included both plenary sessions and discussions in groups of six to eight participants. Unlike standard climate DMPs, participants worked on a range of outputs over the course of the project rather than a single list of policy recommendations. These outputs included a hypothetical party manifesto and a hypothetical ‘green’ business plan.

Data for all activity was recorded online. Where staff and resources allowed, workshop sessions were transcribed verbatim. Otherwise, sessions were live transcribed non-verbatim to capture the main points that each speaker made. BritainThinks carried out a first round of data analysis with input from the authors. This resulted in a report covering the main findings of the research for policy practitioners (Britain Thinks 2022).

### Data analysis

The first round of analysis looked at attitudes towards different forms of climate citizenship. However, it paid less attention to the reasons and arguments offered for these attitudes or how these were shaped by participants’ evolving understandings of the challenges of achieving net zero. The paper’s authors therefore undertook a second round of data analysis to better understand these dynamics.

We followed a grounded theory approach, making few assumptions about the connections between people’s understandings, affective states, and dispositions. Our analysis drew primarily from the deliberative workshops, with online platform data used only to support emerging findings from workshop data. Drawing on the approach of Saldaña (2015), we analysed the data in five iterative phases, moving from the raw data to a coherent set of themes. The five stages progressed as follows. First, we read all the relevant data with a view to identifying statements relevant to the research focus, and clustered statements expressing similar sentiments into codes. Second, we rationalised the initial set of codes from the first pass of coding into a smaller and more coherent set of emergent themes in dialogue between two of the authors. Third, we revisited the data and coded it against emerging themes to test and elaborate them. At this stage we reviewed polling data from the sessions and online-workshops where it helped provide additional evidence to emerging themes. Fourth, we engaged in a reflective process of identifying connections between themes and an emerging ‘bigger picture’ to uncover suggested meanings beyond what was directly said (Saldaña 2015). Finally, we presented the preliminary findings to four researchers not directly involved in the analysis, one of whom had been present during the Net Zero Diary workshops, to test the themes for coherence and plausibility. This way, we constantly moved back and forth between the raw data and emergent themes, in a process similar to constant comparison (Glaser and Strauss 1967).

## Findings

Table 1 provides a summary of the themes and subthemes identified. Theme 1 covers an apparent tendency towards greater pessimism regarding achieving net zero amongst some of the participants, the more they understood about the scale of changes required. Theme 2 covers a tension in the way participants understood the role of the state in the net zero transition, which potentially accounts for some of the observed pessimism. Theme 3 then captures attitudes towards different forms of climate citizenship. In the discussion, we bring these themes into dialogue with each other and the wider literature on emotions, affects and climate citizenship.

**Table 1:** Summary of main themes and subthemes

Theme	Summary
**Theme 1: Tendency for pessimism about achieving net zero emissions by 2050 to increase with awareness of the issues involved**	A higher ratio of participants with more prior engagement on net zero issues started of the process being pessimistic about achieving net zero by 2050. Over the course of the processes, there was also a tendency for increased pessimism amongst some participants.
**Theme 2: There is a constitutive tension between the state needing to act but being unlikely to act**	Government is the actor with the greatest responsibility and capacity to drive the transition to net zero. However, there are strong reasons the state is unlikely to take the necessary actions. This creates a constitutive tensions for thinking about the net zero transition.
Sub-theme 2.1: Tension of the state with regard to individual action	Individuals are unlikely to make the sacrifices necessary to achieve net zero without support and some coercion. The state is the actor with the greatest capacity to provide this support and impose this coercion. No political party would be willing to take such actions as they would provide electorally unpopular.
Sub-theme 2.2: Tension of the state with regard to private business	Private businesses will only support the transition if there is scope to profit from it. This profit motive will not be enough to achieve net zero. The state therefore needs to step in to drive change in the private sector. The interests of political elites often overlap with business elites, and business elites invest in lobbying government. The state is therefore unlikely to force the necessary changes in the private sector.
Sub-theme 2.3: Background distrust in government	Recent political scandals and policy failures have generated a general distrust in government.
**Theme 3: Individual and community-based forms of climate citizenship take priority**	Though the state is identified as the key actor in driving net zero, priority is still given to individual behaviour changes and community level action.
Sub-theme 3.1: Scepticism about directly influencing state decisions	Many participants are sceptical about their capacity to influence state decision-making through voting, lobbying their elected representatives, or taking part in collective action.
Sub-theme 3.2: Faith in the ‘local’ and the ‘communal’	There is strong support for local and communal level activities such as community power schemes; market gardening; local food distribution networks; and local decision making/ participatory governance initiatives.
Sub-theme 3.3: Retreat to the individual	Even having identified the inadequacies of individual behaviour change to drive net zero, participants still highlight individual actions as the most efficacious when asked directly, and want to discuss and share the things they personally were doing to reduce their emissions.

These themes reflect the arguments and reasoning of the whole group as they developed over the course of the process. We use descriptors such as ‘most’ and ‘many’ to highlight attitudes and views expressed the most frequently or that appeared to have the highest level of consent between participants. We have indicated throughout where views were held by a smaller number of people or where dissenting views were expressed. All findings are based primarily on the qualitative analysis. Descriptive statistics are included where these are pertinent to the qualitatively derived themes, however, the relatively small sample size and non-independence of the data precluded statistical analysis. All names in quotes have been changed to protect the anonymity of participants.

### Main theme 1: Tendency for pessimism about achieving net zero emissions by 2050 to increase with awareness of the issues involved

The first theme that emerged was an apparent tendency towards greater pessimism over the course of the process. This indicates that increased awareness and learning about the scale of the net zero challenge can lead to increased pessimism. This link between awareness and pessimism is seen in quotes such as:

*“It just shows you the scale of what's got to be achieved and that makes it more unlikely that we can achieve that by 2050.”***General public in W4S4.**

*“(after reviewing Government Net Zero Strategy) You realise it's nowhere near enough … if you actually learn about Net Zero, you realise there's nothing."***Engaged consumer in W4S5**

Whilst participants were concerned about climate change and had a strong desire to see positive climate action, many believed that time was running out. Indeed, some suggested that the window for action may have already closed. Even whilst some saw the 2050 target for net zero as too late, they simultaneously thought that it would be very difficult to achieve net zero any sooner. Note the following view expressed about the 2050 timeline:

*“I’m an optimist. But it is a struggle to realise how much needs to be done [in contrast to] how much has already been done. 2050 is too late, we will get to a point where we can't backtrack” Alumni in W1S2*

**Fig. 1:**
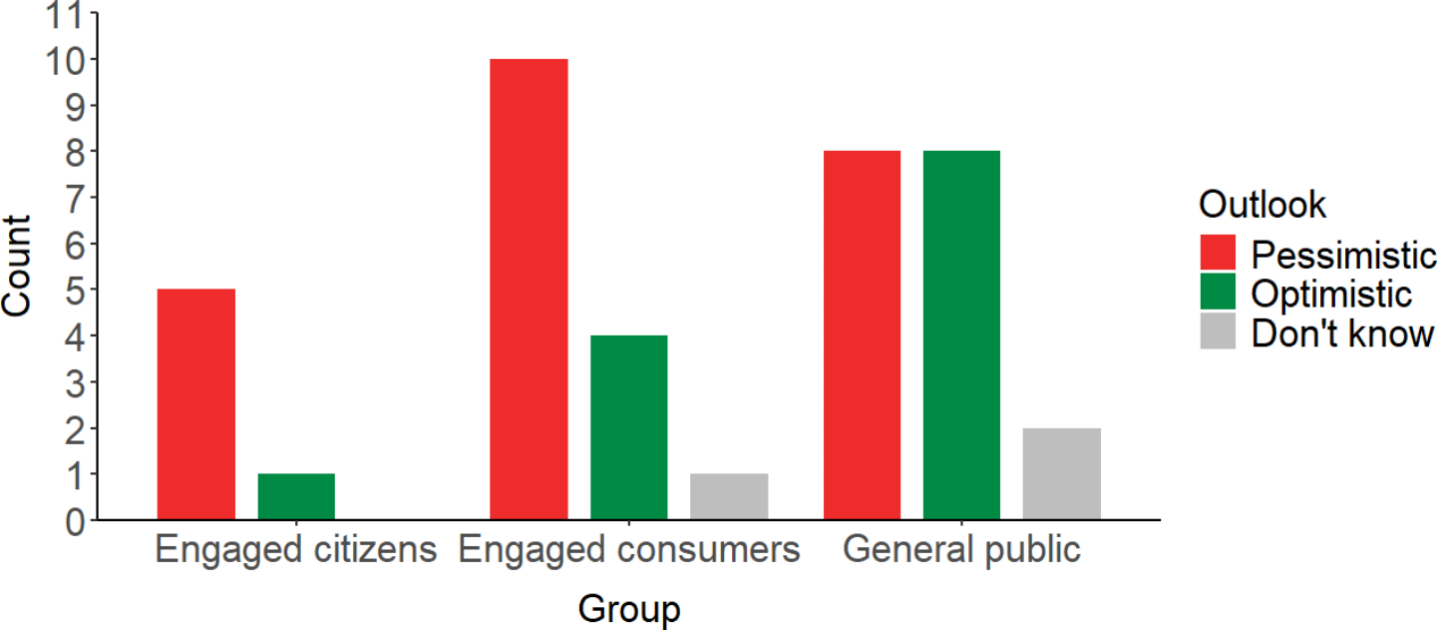
Descriptive statistics to the question in wave 1 online diary “How optimistic or pessimistic do you feel about the UK’s chances of cutting its carbon emissions to become ‘net zero’ by 2050 (the current government target for avoiding dangerous climate change)?” by participant group. ‘Pessimistic’ is an aggregate of responses ‘Very pessimistic’ (*n* = 7) and ‘Fairly pessimistic’ (*n* = 16). ‘Optimistic’ is an aggregate of ‘Very optimistic’ (*n* = 3) and ‘Fairly optimistic’ (*n* = 10)

**Fig. 2:**
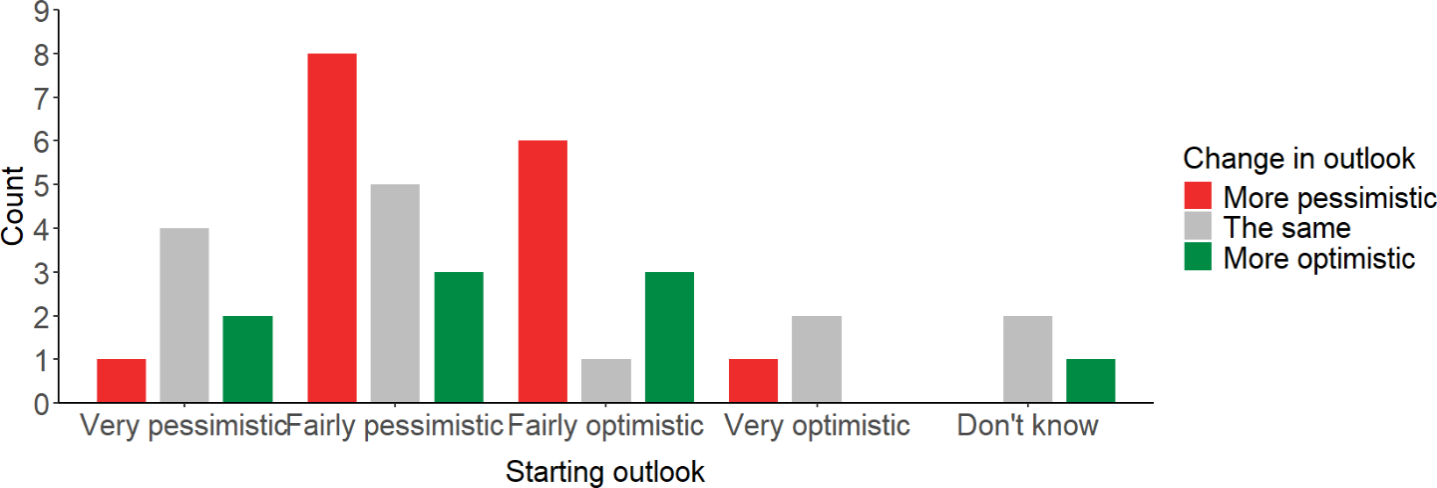
Descriptive statistics from responses to the question in wave 4 online diary: “Compared to before starting the Net Zero Diaries, how do you feel about the UK’s ability to reach Net Zero by 2050?” by starting outlook on chances of reaching net zero. ‘More pessimistic’ is an aggregate of responses ‘A lot more pessimistic’ (*n* = 8) and ‘A bit more pessimistic’ (*n* = 8), and ‘More optimistic’ is an aggregate of ‘A lot more optimistic’ (*n* = 1) and ‘A bit more optimistic’ (*n* = 8)

Descriptive statistics from the polling data from the online platform appears consistent with this connection between awareness and pessimism. Firstly, a greater proportion of those in the ‘engaged citizens’ and ‘engaged consumers’ groups started the process being pessimistic, than those in the ‘general public’ group (Fig. 1). These two groups could be expected to be more aware of the challenges of net zero than the general public at the start of the process. Second, between wave one and wave three, eight people become a lot more pessimistic, and eight a bit more pessimistic about the UK achieving net zero. Eight become a bit more optimistic, and just one became a lot more optimistic (see Fig. 2). Increased awareness and understanding over the course of the plausibly explains the shift towards pessimism over time. Though again we caution that we were not able to statistically test the significance of the differences between groups at the start of the process, or the difference between more optimism and more pessimism later in the process.

### Main theme 2: There is a constitutive tension between the state needing to act but being unlikely to act

**Fig. 3:**
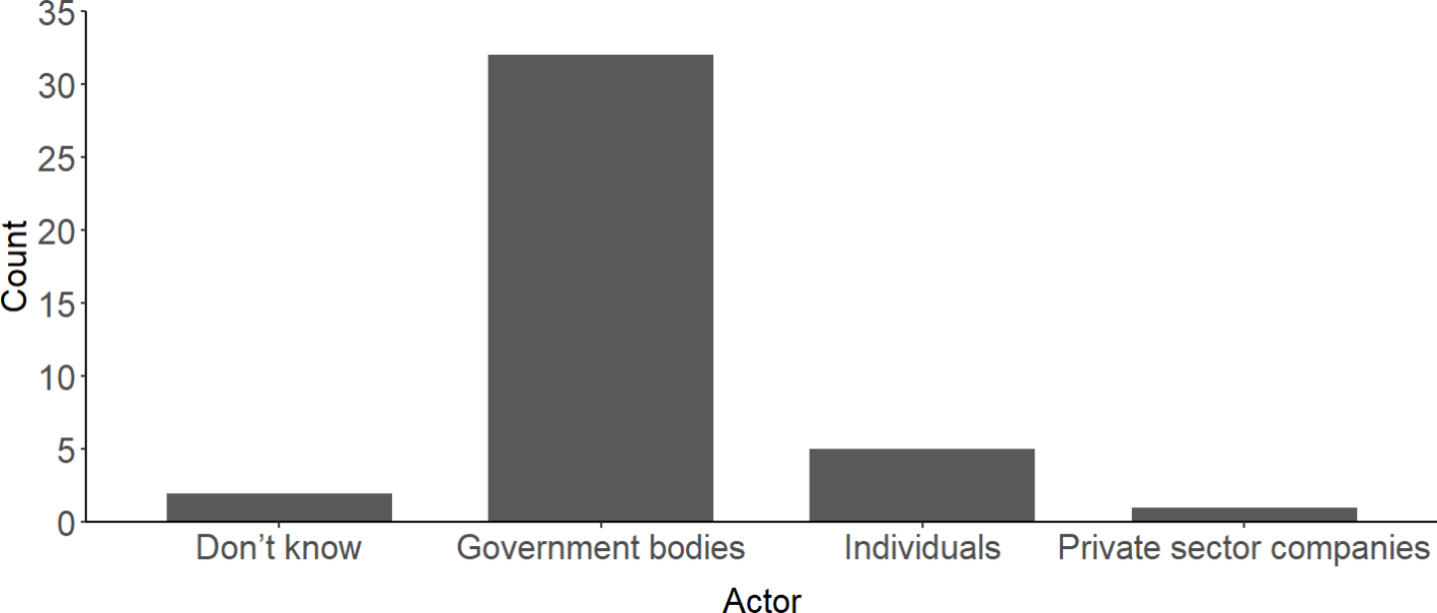
Descriptive statistics from responses to question in wave 4 online diary: “From the following options, please tell us who you think is most responsible for tackling climate change on a global scale?”

Participants spent a lot of time talking about the role of individuals, businesses, and government in the net zero transition (particularly in W1S2, W2S2-4, W2S8, W3S5, W4S3 and W4S6 (see Supplementary Material)). In the first workshop, we asked participants which of these groups they believed bore the most responsibility for net zero objectives. At this stage, many responded by saying that responsibility is shared equally between individuals, businesses, and government. Some maintained this view throughout the NZD process. However, most participants shifted away from this line of argumentation the more they learnt about and reflected on the causes of climate change. They subsequently identified government (referred to here interchangeably as ‘government’ or the ‘state’ to reflect language used by participants) as bearing the greatest responsibility and ability to act (asked directly in W1S1 and discussed extensively in all workshops). This position is further evidenced in polling from the online diary in wave four (see Fig. 3).

However, even whilst participants pointed to government as bearing the greatest obligation to act, many simultaneously believed that the state is unlikely to play the role that they had identified for it. People had well-articulated and coherent reasons for holding this seemingly conflicting position. We explore this below, where we draw attention to what we call a *constitutive* tension that shaped the reasoning of some in the group.

#### Sub-theme: Tension of the state with regard to individual action

Most participants concluded that reaching net zero could not be achieved by placing the onus on individual forms of action. Some felt that popular discourse places too much emphasis on individual action at the expense of state or private sector action (when asked directly about who had the greatest responsibility in W1S1, but also discussed throughout). Others felt that humans are too inherently greedy or selfish to make what might be perceived as significant sacrifices to their standards of living. The view that pro-climate behaviours necessarily involves a strong element of ‘sacrifice’ was implicit in a lot of the discussions. At other points, the view was expressed explicitly.

*“We are doing our bit, most of the public is doing their bit, we’re doing more than we used to do before. It feels like the emphasis has been put on us instead of them (government and companies).” General public in W1S4*

*“I think it’s very distracting, telling people to have short showers and cold showers, and all these tiny little things, recycling, washing out…It takes our eye off the main ball” Engaged consumer in W2S4*

In addition, most people felt that many do not have the financial resources or time to make more pro-climate choices in their lives (this was seen particularly when discussing what government needed to do to support individuals, e.g. in W2S3 and W4S3, and in discussions of fairness, e.g. W2S8).

Consequently, participants’ views converged on a consensus that relying on individuals to voluntarily change their behaviours in the absence of government support is not viable. In this context the state was seen as having a key role in encouraging, providing the resources, and – if necessary – directly forcing individuals to act. Yet, in reaching this conclusion, an added dilemma seemed to emerge in participants’ calculations. Though the state was seen as the main actor to drive individual behaviour change, participants simultaneously expressed serious doubts about whether government would ever embrace this kind of policy programme.

As discussed above, participants believed that the only way to achieve net zero by 2050 would necessarily involve, at least in some instances, ‘sacrifices’ to standards of living. Many thus calculated that government would not do what is needed because of the electoral consequences of doing so (mentioned when discussing the role of government in W2S3 and W4S3, and when discussing the Net Zero Strategy, e.g. in W3S3 and W4S4-5). Participants’ reasoning thus led them to what seemed like a dead end. By carefully piecing together the complexities of climate policymaking, they had ended up in a place where pieces of the jigsaw did not seem to fit together. But this was only the first constitutive tension that participants landed on.

#### Sub-theme: The tension of the state with regard to private business

Initially, attitudes toward the role of private business in the transition were underpinned by an assumption that there was a fundamental conflict between the need to maximise profit and the prioritisation of sustainability (W3S5). This led to scepticism that larger corporations could play a positive role in the net zero transition, which was exacerbated by concerns over ‘greenwashing’.

“*Profits! They’ll never do this because it will impact on the profits. Just look at Grenfell, its consumer capitalism. It keeps its face hidden, but you see its claws sometimes*.” ***Engaged consumer in W2S3***

As discussions progressed, this view became more nuanced. Participants came to the view that there are instances where the net zero transition aligned with business opportunities (throughout workshop three). For example, the growth of electric vehicles was seen as an area where businesses are driving change towards a net zero economy. One participant noted that this alignment is not a result of businesses prioritising climate concerns, but rather a result of car companies seeing a profit opportunity in electric vehicles. This attitude towards larger corporations fed into how participants thought about potential future ‘green jobs’ in the net zero transition. Several participants expressed concern that green jobs provided by private businesses may be similar to many existing jobs in the UK economy, in that they may be low paid and offer poor working conditions.

*‘They’re there to make money and satisfy their shareholders. If that means being green then they’ll do that. But a lot of what means being green in part A can mean being much less green in part B. So they use one type of fuel that cuts down on CO2 at one stage but adds to it later on. That isn't part of the equation. It’s terribly difficult to tell how green something is*.*’ General public in W3S5*

In areas where participants believed there is a conflict between profit and climate goals, many participants stated that government should compel large businesses to prioritise climate goals over profit. Views on how this could be achieved varied, with some supporting nationalising businesses and others suggesting a regulatory approach.

Yet here again, there was significant scepticism that the state would play the role participants had identified it should. One key perceived barrier was the significant overlap between political and business elites, resulting in both groups sharing a set of material interests (expressed when discussing the role of government, and particularly the capacity of civil action to influence government, e.g. W2S4). Participants mentioned recent controversies in the UK over contracts being handed out to politically connected businesspeople during the Covid-19 pandemic. Others pointed to instances of MPs sitting on the boards of companies or drew attention to businesses investing in lobbying activities to ensure they were not regulated in ways that limited their profit maximising potential.

Thus, as with the relationship between individuals and the state, participants had strong reasons for identifying the state as the necessary agent to drive the transition to net zero in the private sector, whilst simultaneously believing that the state would be unlikely to take the necessary actions.

#### Sub-theme: Background distrust in government

The specific mistrust that the state would fulfil its necessary role in the net zero transition in relation to individuals and the private sector was coupled with a wider, more general, mistrust of the state (expressed throughout the sessions). Several reasons were given for such an attitude. These included recent political scandals mentioned above related to the Covid-19 pandemic, as well as prior policy failures. One participant mentioned that they worked on an initiative in their youth called ‘Health for all by 2000’, and noted the irony of currently experiencing a global pandemic. A more recent policy failure mentioned frequently was the ‘Green Homes Grant’, a now defunct and widely criticised initiative to increase the installation of energy efficiency measures in homes (PAC 2021).

‘*We don’t really trust the government, they’re always lying to us, so the trust is gone and people are quite sceptical’. Alumni in W4S2*

Although party preferences were expressed during a task looking at party manifesto pledges, participants did not typically make a significant distinction between the major parties when discussing the likelihood of the state driving the net zero transition.

### Main theme 3: Individual and community-based forms of climate citizenship take priority

As discussed, most participants saw the state as the actor with the greatest responsibility and capacity to drive the net zero transition. We might therefore expect that they would prioritise or support direct attempts to influence state decision making by voting or through other political activities. In fact, the opposite appeared to be true. Participants were generally sceptical about efforts to engage in formal politics around the issue of climate change. They were more supportive of local or community scale activity, and in making behaviour and consumption changes in their own lives.

#### Sub-theme: Scepticism about directly influencing state

Participants rarely sought to distinguish between the major parties when discussing the state. There was, potentially relatedly, also very little discussion about the importance of voting as an approach to influencing climate policy development. Similarly, there was relatively little support for attempting to communicate with or lobby elected representatives at the national level (seen through polling on most effective actions, discussion in W2S4, and W4S6 on how the public should be included in net zero conversations). Some participants recalled instances when they or other people they knew had attempted to contact their Member of Parliament but not received a response. This seemed to fuel mistrust in parliamentarians.

In general, many participants felt disempowered and unable to affect any change at the scale of national politics. This feeling came to the fore in discussions about the possibility of different, more participatory forms of policy making (W4S6). Though unsure about the specific institutional design of such changes, there was broad based support for opening up more opportunities for citizens to be heard by government.

*“I agree with Patricia that there’s not a lot that individuals in this country can do. We can give our input but it’s not going to make much of a difference.” General public in W2S3*

Participants had ambivalent views about the ability of protests, social movements, and other forms of action outside of formal political channels. Only a small number of participants said that they had taken part in these kinds of activities or that they were planning to. Political action outside of institutional mechanisms were also accorded low priority when participants were asked to list the forms of action on climate change that would be most effective. During the workshops, participants discussed the role of groups such as Extinction Rebellion and Insulate Britain (W2S4). Many participants were broadly supportive of these groups, though some questioned the use of tactics that disrupt people’s lives. A number of participants mentioned that social movements had been necessary for driving state action in the past, but did not believe that current mobilisation around climate change was of a scale that could produce meaningful change.

#### Sub-theme: Faith in the ‘local’ and the ‘communal’

Throughout the process many participants voiced strong support for ‘local’ and ‘communal’ approaches to climate citizenship. This was expressed in support for and/or participation in activities including: community power schemes; market gardening; local food distribution networks; and local decision making/ participatory governance initiatives. When asked to design their own ‘sustainable business’, all break-out groups designed a small, locally oriented not-for-profit organisation (W3S8).

This support for the small and local was motivated by different factors. There was an implicit assumption amongst many participants that small scale forms of production were more likely to adopt sustainable practices than larger corporations. Some older participants also expressed nostalgia for a time when community cooperation was perceived to be higher and private business were orientated more towards serving local communities.

*“I’d just like to add my support to what Dave and Andrew are saying about localism. I don’t think there’s enough emphasis put on localism. It ticks a lot of boxes; it cuts down on transportation and the problems that brings, it can bring local and nourishing products. I think localism is really underrated.” Alumni in W1S3*

This orientation towards the local appeared to be a source of hope for some participants. This may explain some of the participants not exhibiting an increase in scepticism over the course of the process.

*“You have to give people stuff that they can influence. If you take small steps as a time, people see the benefits and get more engaged. So starting locally and thinking about what people can do in their house, with their lifestyle...there has to be a benefit. We started at a basic level of not knowing what Net Zero meant, and now it has evolved. And I think that’s the way to look at it, from a community perspective.” Engaged consumer in W4S6*

#### Sub-theme: The retreat into the individual

Despite voicing frustration that individual action is so prevalent in popular climate discourse, many participants still prioritised individual behaviour changes when asked to list actions with the highest impact. Perhaps more revealingly, participants spoke most passionately when discussing the consumption choices they were making in their own lives. Participants clearly took pride in the individual actions they were taking to reduce their personal emission footprint and felt that it was important to let others know about these.

Tellingly, this view – the prioritisation of individual consumptive acts over other forms of action – did not appear to shift once participants had discussed the role of the individual in relation to the state and private business in more detail. The valorisation of individual action therefore persisted alongside a purported belief in the priority of state action and the inadequacy of individually motivated behaviour change. Hence, we labelled this code as a ‘retreat’ *into* the individual. As such, it does not appear that this view was held because people believe individual consumption choices to be the most efficacious form of climate action. Rather, it appears that people felt individual action is among the limited possibilities available to them to enact some form of change.

## Discussion

### Interpreting our findings: individual action from ‘reflexive impotence’

At first glance, participants’ attitudes towards different forms of climate citizenship appear discordant with the normative view that participants held about the roles and responsibilities of different actors in the net zero transition. Though the state was routinely identified as the actor with the greatest responsibility and capacity to act, participants showed little interest in and support for politically oriented forms of climate citizenship. Conversely, though individually motivated behaviour change was identified as insufficient for achieving net zero, individual consumption choices were frequently prioritised during diary tasks and workshops. Support for small scale enterprise and consumption of local produce make sense in light of distrust of large corporations. Though again, when discussing the private sector in detail, it was state intervention that was identified as the key lever for driving change, not a shift in individual consumption towards SMEs and local business.

Attitudes towards different forms of climate citizenship make sense only in light of the tension of state action/inaction participants expressed. More politically oriented forms of climate citizenship must be underpinned by a belief that such action would be effective – that is, people must have a sense of political efficacy (Nabi et al. 2018). Those in our sample appeared to exhibit relatively low feelings of political efficacy, due to a generalised distrust in the state, coupled with an understanding of barriers to state action specific to the climate crisis.

Prior work has identified that political distrust is linked to lower support for environmental policies, particularly policies such as environmental taxes (Fairbrother 2017). Publics can be concerned that policies will be poorly implemented, or funds from environmental taxes could be misspent. This can make publics less likely to call for policies that, if trust levels were higher, they might support. This can explain why concern for climate change does not necessarily translate in support for specific policies to address climate (Ipsos and CAST 2022). For our sample, the concern was less that states would implement policy poorly, than that they would not implement sufficient policy at all. The effect in terms of climate citizenship is potentially similar—that publics will make fewer demands on policy makers for climate policies than they might otherwise.

The valorisation of the small, the local, and the individual in our study can be interpreted not as resulting from belief in their efficacy, but because more politically orientated forms of citizenship are typically seen, or at least felt, to be futile. Importantly, it is not necessary for this full line of reasoning to be conducted by everyone. People’s attitudes towards different forms of climate citizenship are as likely to be shaped by broad feelings and affective states as to be explicitly logically deduced. What is important is that people feel more positive towards more locally and individually oriented forms of climate citizenship.

A commitment to individual action should be understood then not as illogical, but as a grasping for hope and agency in the face of a seemingly intractable problem. In cultural theorist Mark Fisher’s terms, it can be seen as a form of ‘reflexive impotence’ (Fisher 2012). Fisher coins this term in trying to explain the lack of political activism amongst teenagers in the UK in the face of declining prospects and material circumstances. In Fisher’s words, “they know things are bad, but more than that, they know they can’t do anything about it” (p. 21).

In other contexts, it has been noted that a decrease in trust in government can lead to higher levels of perceptions of environmental risks. This in turn can lead to greater levels of political activism, at least in countries with relatively open political climates (Verner 2023). This may explain the upsurge in climate protest movements in the UK and elsewhere, particularly amongst younger generations. However, at least for our sample, distrust did not appear to translate into intention to participate in more active forms of political participation. The key mediating factor is likely feelings of political efficacy amongst different demographics. Low levels of trust typically correlate with low feelings of political efficacy, which in turn correlate with lower levels of political participation (Osborne et al. 2015; Prats and Meunier 2021; Dirksmeier and Tuitjer 2022). Environmental concern can motivate behaviours such as signing a petition. However, in the absence of feelings of political efficacy, such concern does not typically motivate more active forms of citizenship such as attending marches. On climate, there is evidence that perceived ability to influence government doubles the odds of participating in environmental marches (Boulianne and Ohme 2021).

Another possible interpretation of our findings on attitudes to different forms of climate citizenship is that, even if they expressed concern for climate change, it was simply not a high enough salience issue to motivate politically active forms of climate citizenship. Support for individual action and forms of local and community engagement could be, on this view, the first steps on a pathway towards becoming more politically active if the issue gains salience and people notice the limitations of their existing forms of engagement. Though there is some support for such a ‘spill over’ effect of small behavioural changes in the literature, the evidence is mixed (Nash et al. 2017; Nash et al. 2019). Regardless, we believe it is compatible with the explanation we offer above. It is possible that individual and local action can act as a gateway to more political forms of climate citizenship, whilst still being true that a sceptical attitude towards the likelihood of state action blocks many people from progressing along the path.

Importantly, the tension of the state that emerges from our data cannot be seen as purely an issue of perception. The importance of the profit motive in shaping private sector engagement with the net zero transition is well documented (Lamb et al. 2020; Christophers 2021; Franta 2021). Whilst electric vehicle uptake is accelerating with the backing of all major car manufacturers, efforts towards modal shift in transport lag behind (Climate Change Committee 2023; International Energy Agency 2023). Similarly, though the growth of renewable electricity generation is accelerating, incumbent energy companies are still slowing down the energy transition due to the higher profits available from continuing to supply fossil fuels (Christophers 2021). The essential role of the state in driving the transition is therefore largely uncontested.

At the same time, no major economy has introduced policies sufficient to deliver on the Paris Agreement target of limiting emissions to 1.5 °C. This is at least in part due to the influence of the private sector on state decision making. Such influence can take many forms, such as straightforward corruption and profiteering by politicians, conflict of interests for politicians with positions or financial stakes in the private sectors, dependence on certain sectors for state revenue and financial stability, and aggressive lobbying efforts from the private sector (Conway and Oreskes 2012; Clapp 2014; Buller 2020; Lamb et al. 2020; Franta 2021; Stoddard et al. 2021).

In terms of individuals and the state, it is an oversimplification to suggest that people will not accept climate policies that inconvenience them or force them to give up existing lifestyles or consumption habits. Evidence suggests that what is crucial is how the question is framed; initial pushback against climate policies that are seen to impose additional costs can be tempered if policies are perceived as fair, effective, and to produce co-benefits (Bain et al. 2015; Reynolds et al. 2018, Whitmarsh et al. 2019). It is certainly true that elected representatives perceive this to be the case, and as a result are hesitant to bring in climate policies targeting individual consumption patterns (Westlake and Willis 2023).

Our participants intuited these contradictions relatively quickly as they learnt about the intricacies of climate policy. This suggests that such views could easily become widespread in the population as people’s awareness and understanding of climate policy grows (if they are not already). They should therefore be a serious consideration for those trying to promote more active forms of climate citizenship.

### Implications for building climate citizenship

For those interested in fermenting a democratic response to the climate crisis, there is an unfortunate irony to our findings. The tension of state action, whilst motivating more individualistic and local forms of climate citizenship, likely requires more politically oriented forms if it is to be resolved. Active citizenship is likely a key means by which some of barriers to state action identified can be overcome (Agnone 2007; Muñoz et al. 2018; Chan et al. 2020; Laybourn et al. 2021).

As Fisher discusses when introducing the term, ‘reflexive impotence’ can become a self-fulfilling prophecy. Resigning oneself to the idea that nothing changes makes it more likely nothing will change. Breaking this self-perpetuating cycle and rebuild feelings of trust and efficacy is likely necessary for encouraging more politically engaged forms of climate citizenship. There is no simple answer for how this can be achieved. Political distrust, and associated feelings of low political efficacy, are the consequence of long-term policy failure and thus not easily reversed (McLaren 2012; van der Meer and Zmerli 2017).

Unfortunately, some known mechanisms for building political efficacy are primarily engaged in by people who are not entirely alienated from politics. For example, participation in social movements is known to increase feelings of political efficacy. However, those with higher levels of efficacy are also more likely to participate in social movements to start with (Shafi and Ran 2021). Such participation can also be a double edge sword. Evidence from the Umbrella Movement in Hong Kong suggests political efficacy can decline amongst participants in social movements if they perceive the government as being unresponsive to their demands (Chan et al. 2020). Importantly though, much work on social movements, especially on climate, focuses on younger generations and students. It is known that feelings of political efficacy are already higher amongst more educated, non-married, city dwellers from higher social classes– the typical demographic of students (Battershill & Kuperman, 2023; Morkevičius et al., 2020). This is therefore less likely to be an avenue for developing more active forms of climate citizenships amongst demographics who are not already disposed to taking part in social movements.

Similarly, engagement in forms of public deliberation and participatory policy making can increase feelings of political efficacy (Ergenc 2014). This is a potential benefit of the recent upsurge of climate juries and climate assemblies (Ainscough and Willis 2024). However, here again, the same issue repeats itself. Those with pre-existing higher feelings of political efficacy are more likely to take part in participatory policy making initiatives in the first place (Lebrument et al. 2021).

Social movements and participatory policy making are therefore important for building wider levels efficacy needed for more politically oriented climate citizenship. However, there is a risk that they do not capture demographics who are currently most alienated from active forms of political engagement. This echoes concerns recently raised by Kevin Elliott, that a focus on more demanding forms of political engagement risks overlooking the huge number of people who don’t see any benefit to finding time for such activities (Elliott 2023). There is a need for strategies to counteract a potential lapse into reflexive impotence on climate change amongst parts of the population who are unlikely to take part in social movements or participatory policy making processes. This places a greater emphasis on forms of political communication and on the trust building potential of good policy making.

Political leaders, if interested in building forms of active climate citizenship, must communicate about and be able to demonstrate effective statecraft. Political distrust comes in part from views about corruption, procedural fairness and inclusiveness, and government performance in economic terms (van der Meer and Zmerli 2017). This suggests several avenues for political leaders interested in building forms of active climate citizenship. For example, it is known that hearing about participatory policy making processes can increase feelings of political efficacy (Knobloch et al. 2019). Thus, there may be a role for policy makers communicating more about how they are engaging the public on climate issues. It is also likely that climate policies focused on materially improving people’s lives will help to build political trust. Finally, there is growing evidence that symbolic high-carbon behaviours such as frequently flying can undermine the public’s trust in political leaders to deliver climate action (Westlake 2022). Though small in their overall contribution to emissions, reducing such actions is a vital part of overcoming public scepticism that the state can play its essential role in the net zero transition.

NGOs and policy organisations looking to rebuild feelings of political efficacy around climate change could look to highlight the growing evidence that social movements can and do bring about substantial policy and society level changes (Agnone 2007; Muñoz et al. 2018; Chan et al. 2020; Laybourn et al. 2021). To our knowledge, the efficacy of such communication strategies for increasing feelings of political efficacy is not well understood. This represents a possible avenue for future research.

### Implications for future climate DMPs

Deducing, or at least intuiting, the tension of state action/inaction in the net zero transition leads, by implication, to the conclusion that achieving rapid decarbonisation is unlikely. This conclusion could explain the observed increased in pessimism about achieving net zero amongst some of our participants. Though we have no direct evidence that it is the case, a plausible interpretation of our findings is that many people fundamentally do not believe that the climate crisis is going to be addressed. This raises potential challenges both for the running of climate DMPs, and for attempts to invigorate active forms of climate citizenship necessary for a democratic response to the crisis.

Many critiques of DMPs have been mounted over the years, leading to a much more nuanced understanding of their strengths, weaknesses, and potential uses in the policy process (Curato et al. 2020). One critique that our findings bear on comes from Roslyn Fuller (Fuller 2019). Fuller argues that, as many DMPs are advisory, citizens are not in a position of having to directly confront the consequences of their decisions. For Fuller, this fact has a serious bearing on the way that citizens reason within a DMP, which should lead us to be sceptical of applying their findings and arguments in the creations of actual policy. If Fuller is correct about this point, then those commissioning and designing climate DMPs may need to do more to uncover and explore participants attitudes of hope and pessimism regarding the climate crisis. If participants become more pessimistic about addressing the crisis, and the possibility of state action in particular, as they learn over the course of a DMP, it is possible their reasoning with regards to policy action becomes increasingly detached from real world considerations. This is no reason to dismiss the findings of climate DMPs, but points to a potentially unexplored area of research that could usefully inform the design of future processes.

### Limitations and future research

Our central thesis, that a constitutive tension in beliefs about the role of the state condition and constrain people’s attitudes towards different forms of climate citizenship, has only been inferred from our results and was not explored explicitly with participants. This thesis could be tested and refined through future deliberative work that addresses such dynamics explicitly. Larger sample size studies, using a mixture of small group work and polling, could also help to shed light on how the state features in people’s thinking about forms of climate citizenship. Our limited quantitative data and survey design precluded us statistically testing the apparent link between awareness and pessimism. Future studies, particularly those using survey experiments, could help to further unpack the relationship between these two variables.

## Conclusion

In our study we have attempted to use the learning process of a climate DMP to understand how attitudes to different forms of climate citizenship are shaped. We found an apparent tendency towards increased pessimism regarding achieving net zero the more people learn about the challenges involved, though this is not universal. A key mediating factor may be levels of distrust people have in the state to take the necessary steps to achieve decarbonisation, coupled with a recognition that no other actors can play this role. We suggest that this analysis militates against people gaining a feeling of political efficacy and engaging in active forms of climate citizenship. Future efforts to motivate more active forms of climate citizenship must therefore consider how people’s current and prior experiences of engaging with the state shapes their feelings of political efficacy.

## Electronic supplementary material

Below is the link to the electronic supplementary material.

Supplementary Material 1

## Data Availability

The datasets analysed during the current study are available from the corresponding author on reasonable request.

## Footnotes

1 We are grateful to the energy company Ovo, the environmental NGO WWF, and the public information organisation Citizens Advice. These three organisations funded the project.

2 This label is not meant to reflect current levels of engagement on climate issues, but the fact that these individuals began the process having already received significant information about climate change and the net zero transition.
