# Supplementary material for: Distrust and reflexive impotence in the net zero transition: findings from a longitudinal deliberative mini-public
Source: Clim Change. 2024 Oct 28;177(11):160. doi: 10.1007/s10584-024-03806-2 (PMC11519151; doi:10.1007/s10584-024-03806-2)
Supplement: Supplementary file 1 — Supplementary Material 1 [file 10584_2024_3806_MOESM1_ESM.docx]

**Supplementary material**

**Table 1. Make up of the panel**

|  | **Sample** | **National** |  | **Sample** | **National** |
| --- | --- | --- | --- | --- | --- |
| **Age** | | | **Gender** | | |
| **18 - 24** | 5% | 8% | **Male** | 51% | 49% |
| **25 - 34** | 15% | 14% | **Female** | 49% | 51% |
| **35 - 44** | 5% | 13% | **Social grade** | | |
| **45 - 54** | 38% | 13% | **AB** | 36% | 23% |
| **55 - 64** | 18% | 13% | **C1C2** | 46% | 54% |
| **65 - 74** | 15% | 10% | **DE** | 18% | 23% |
| **75 - 84** | 3% | 6% | **Climate concern** | | |
| **Ethnicity** | | | **Not at all concerned** | 5% | 3% |
| **Asian** | 22% | 9% | **Not very concerned** | 8% | 11% |
| **Black** | 3% | 4% | **Fairly concerned** | 41% | 41% |
| **Mixed** | 3% | 3% | **Very concerned** | 46% | 44% |
| **White** | 76% | 82% | **Don't know** | 0% | 2% |
| **Other** | 0% | 2% |  | | |

**Table 2. Overview of workshop activities**

| **Workshop 1. Individuals and net zero**: The concept of net zero; emissions from food, transport and energy; and individual actions diarists were already taking | | |
| --- | --- | --- |
| **Main sessions** | **Main activities** | **Code** |
| Introduction to climate change | - Polling on attitudes to climate change, net zero target and responsibilities of different actors - Breakout group discussions | W1S1 |
| Exploration of net-zero | - Video introducing concept of net zero - Plenary discussion - Breakout group discussions - Presentation on structural constraints to individual action | W1S2 |
| Emissions from food | - Expert presentation - Question and answer in plenary - Breakout group discussions | W1S3 |
| Emissions from transport | - Expert presentation - Question and answer in plenary - Breakout group discussions | W1S4 |
| Emissions from energy | - Expert presentation - Question and answer in plenary - Breakout group discussions | W1S5 |
| Wrap up | - Polling questions on level of optimism of achieving net zero | W1S6 |
| **Workshop 2. Government and net zero**: Discussing the role of government in reaching net zero, and how the concept of fairness related to net zero | | |
| **Main sessions** | **Main activities** | **Code** |
| Introduction | - Facilitator presentations about structure and aims of the workshop | W2S1 |
| Government and net zero | - Introduction to the topic - Themed breakout group discussions   - The roles and responsibilities of government   - How can government change individual behaviour?   - The role of civic action in influencing government | W2S2 |
| Party Manifestos | - Plenary presentation of anonymised party manifesto commitments on climate - Breakout group discussion - Polling questions on attitudes towards each manifesto | W2S3 |
| Fairness and climate change | - Facilitator led plenary presentation - Videos from Turn2Us, Greenpeace, and CarbonBrief on topic of fairness - Themed breakout group discussions   - International fairness   - Domestic fairness   - Intergenerational fairness | W2S4 |
| Manifesto building | - Designing climate manifesto in breakout groups - Plenary presentation on each manifesto - Polling to vote for manifestos | W2S5 |
| **Workshop 3. Business and net zero**: the role of business in achieving net zero; different business models; outcomes of COP26 | | |
| **Main sessions** | **Main activities** | **Code** |
| Introduction | - Facilitator presentations about structure and aims of the workshop - Discussion of initial reactions to COP26 outcomes | W3S1 |
| Views on financial commitments at COP26 | - Breakout group discussion | W3S2 |
| Views on gas boiler phase out and the UK Net Zero Strategy | - Breakout group discussion | W3S3 |
| Views on call for action on small island nations at COP26 | - Breakout group discussion | W3S4 |
| Reflections on the role of individuals and businesses | - Breakout group discussion | W3S5 |
| Understanding sustainable business models | - Plenary expert presentation on different models for sustainable businesses - Breakout group discussions on presentation | W3S6 |
| Business showcase | - Presentations from representatives of different businesses - Question and answer session | W3S7 |
| Sustainability plan co-creation | - Designing sustainable business in breakout groups - Presentation of ideas to panel of experts in plenary | W3S8 |
| **Workshop 4. Bringing it all together:** support individuals need from business and government; the Government’s Net Zero Strategy | | |
| **Main sessions** | **Main activities** | **Code** |
| Introduction | - Facilitator presentations about structure and aims of the workshop | W4S1 |
| Reflections on process so far | - Breakout group discussion | W4S2 |
| What government and business need to do to support people making changes | - Facilitator presentation on how business and government could help - Breakout group discussion | W4S3 |
| Responses to the UK Government Net Zero Strategy | - (pre reading on Net Zero Strategy) - Facilitator led presentation based on Climate Change Committee review of Net Zero Strategy - Breakout group discussion | W4S4 |
| Expert views on Net Zero Strategy | - Expert panel discussion on strengths and weaknesses of Net Zero Strategy - Question and answer session with experts - Sector specific breakout groups for discussion with expert   - Energy   - Transport   - Food | W4S5 |
| How should public be included in Net Zero conversations in future? | - Breakout group discussion | W4S6 |
